# Validation of the sunlight exposure diary and the erythropoietic protoporphyria impact questionnaire (EPIQ)

**DOI:** 10.1186/s13023-025-04012-8

**Published:** 2025-09-30

**Authors:** Hetanshi Naik, Susan D. Mathias, Michelle P. Turner, Megan O’Grady, Chelsea Norregaard, Hilary H. Colwell, William Savage, Melanie Chin

**Affiliations:** 1 Department of Genetics, Stanford University, Stanford, CA USA; 2 Health Outcomes Solutions, Palm Beach Gardens, FL USA; 3 Disc Medicine, Watertown, MA USA

**Keywords:** Erythropoietic protoporphyria, X-linked protoporphyria, Quality of life, Patient-reported outcomes

## Abstract

**Background:**

Erythropoietic protoporphyria (EPP) and X-linked protoporphyria (XLP) are rare disorders that can negatively affect one’s health-related quality of life (HRQoL) because of pain from phototoxic reactions and the avoidance of sun exposure that is recommended. There is a need for reliable and valid patient-reported outcome measures (PROMs) that address all aspects of EPP/XLP, including symptoms and impacts. Our objective was to assess 2 recently developed PROMs, the Sunlight Exposure Diary and the EPP Impact Questionnaire (EPIQ), to determine their factor structure and explore their psychometric properties.

**Results:**

During a clinical trial conducted from January 2023 to August 2024 evaluating an oral therapy to improve sunlight tolerance in adults, participants with EPP or XLP completed the Sunlight Exposure Diary, the EPIQ, and other PROMs (PROMIS-57 v2.1, PROMIS Short Form v2.0 - Social Isolation, and PROMIS-Neuropathic Pain Quality v2.0 scales), at multiple time points via electronic data capture. Data from all treatment groups were combined for analysis. 65 participants with a baseline and at least 1 follow-up assessment on the EPIQ were included (mean age = 45, 51% male, whole-blood metal-free PPIX levels = 9335.5 µg/L). Exploratory Factor Analysis identified 1 underlying factor in the Sunlight Exposure Diary (“Tingling/Itching”) and 3 in the EPIQ (“Duration of Full Reaction,” “Overall Change,” and “Overall Severity and Impact”). The factor from the Sunlight Exposure Diary showed less consistent performance. A single item from the Sunlight Exposure Diary was also evaluated (the Daily Daylight Tolerance Over 2-Week Interval). The 3 factors from the EPIQ demonstrated acceptable to strong psychometric properties in terms of reliability (internal consistency, test-retest), validity (construct, known groups), and responsiveness. Ranges for meaningful change, using anchor- and distribution-based approaches, were established.

**Conclusions:**

These PROMS address the need for EPP/XLP-specific measures that assess the duration, severity, and impact of early warning symptoms and full phototoxic reactions, and capture impacts of EPP and XLP on well-being and HRQoL. Results suggest that the PROMs are reliable and valid, supporting their use in future research and relevant for assessing the experiences of individuals with EPP or XLP.

**Supplementary Information:**

The online version contains supplementary material available at 10.1186/s13023-025-04012-8.

## Background

Erythropoietic protoporphyria (EPP) and X-linked protoporphyria (XLP) are rare genetic disorders characterized by the accumulation of protoporphyrin IX (PPIX) in erythroid cells [1, 2], leading to severe phototoxicity [3] and possible liver complications [2, 4, 5]. EPP (and XLP) most commonly begins in infancy or childhood [1] and negatively affects health-related quality of life (HRQoL) because of pain from phototoxic reactions and avoidance of sun exposure, leading to impediments of social and physical functioning [6–8].

Patient-reported outcome measures (PROMs) are important tools for collecting the patient perspective and can provide information that improves patient-clinician communication and ultimately leads to better health outcomes [9, 10]. PROMs can help quantify patient burden, assess changes in health status, and evaluate the effectiveness of treatments from the patient perspective.

Global PROM tools have been used to assess HRQoL in EPP/XLP, but do not always capture impacts specific to EPP/XLP, such as how individuals are affected by coping with EPP/XLP and the need to avoid situations when they may experience phototoxic reactions [11]. Tools developed for dermatologic conditions or EPP specifically, like the Dermatology Life Quality Index (DLQI) and EPP-QoL, fail to assess severe pain or the avoidance of sunlight [11, 12]. Additionally, the EPP-QoL’s “well-being” domain was removed during empirical validation due to poor psychometric properties [13]. Therefore, there is a need for valid and reliable PROMs that address all aspects of EPP and XLP, including symptoms and impacts.

The Sunlight Exposure Diary [14] and the EPP Impact Questionnaire (EPIQ) [12] are recently developed PROMs for capturing the experiences of those with EPP or XLP. They were developed using patient input as recommended by the Food and Drug Administration in guidelines for PROM development [15–18], and are specific to the experiences of those with EPP or XLP. These PROMs were shown to be content valid; therefore, this study sought to further assess the tools by identifying the factor structure using exploratory factor analysis (EFA) and exploring their psychometric properties, including validity, reliability, responsiveness, and what constitutes meaningful change.

## Methods

### Study population

This study utilized data from individuals participating in the AURORA trial (DISC-1459-201; NCT05308472), a Phase 2, randomized, double-blind, placebo-controlled study designed to evaluate the safety, tolerability, and efficacy of bitopertin in adults with EPP or XLP [19]. Bitopertin, a GlyT1 inhibitor, was investigated for its potential to reduce PPIX levels and improve sunlight tolerance. HRQoL was assessed with the EPIQ, Patient-Reported Outcomes Measurement System (PROMIS)-57 v2.1, PROMIS Short Form v2.0 - Social Isolation, and PROMIS-Neuropathic Pain Quality v2.0 scales. The trial ran from January 2023 to August 2024.

For the current study evaluating the psychometric properties of the Sunlight Exposure Diary and EPIQ, all AURORA trial participants with a baseline assessment and at least 1 follow-up assessment on the EPIQ were included (regardless of the existence of data from the Sunlight Exposure Diary). Trial participants self-administered the Sunlight Exposure Diary and EPIQ. Data from all treatment groups were combined, and all analyses were conducted without regard to treatment assignment.

### Measurements and data collection

The Sunlight Exposure Diary [14] recorded daily sunlight exposure and any resulting symptoms. Participants detailed the total time spent in sunlight, noted any reactions such as “warning” (i.e., prodromal) symptoms or phototoxic reactions, and rated pain severity on a scale from 0 (no pain) to 10 (worst pain imaginable). The diary included a checklist of 12 symptoms (tingling, burning, itching, stinging, sensitivity to hot or cold, sensitivity to touch, pain, feelings of warmth, swelling, redness/discoloration, burst blood vessels, and blisters) that participants could indicate as having experienced along with a severity rating of mild, moderate, or severe for each symptom. The average responses around the 2 weeks closest to target study Days 1, 43, 71, and 121 were used in the analyses. A 2-week window was used to provide a stable and representative measure of the typical experiences of participants because of day-to-day variability in sunlight exposure and symptom reporting, selective sunlight challenges by participants, and missing data from incomplete diary entries.

The EPIQ [12] assessed the impact of EPP/XLP over the past 7 days, including the occurrence of any phototoxic reactions, duration of sun exposure before a reaction, time to improvement and complete resolution, impact on daily activities, and overall quality of life compared to someone without EPP/XLP using a 5-point Likert scale. It also included 3 Patient Global Impression of Severity (PGI-S) items and 5 Patient Global Impression of Change (PGI-C) (from the start of the study) items, rated on 5- or 7-point Likert scales. The PGI-C items were collected only at follow-up visits on Days 43, 71, and 121, as they assess change from baseline.

### Statistical analyses

#### Individual item review and rescaling

Individual item distributions were reviewed to identify the existence of patterns that may introduce bias to the analysis, such as highly skewed responses or floor/ceiling effects. When necessary, items were reverse-scored so that higher scores always indicated better outcomes and rescaled to a 0 to 100 range.

#### Exploratory factor analysis

EFA was conducted to identify relationships between observed variables and their underlying latent constructs. We conducted EFA instead of confirmatory factor analysis (CFA) because the questionnaire was newly developed, and we aimed to explore its factor structure without imposing a predefined model. Using this approach, potential constructs and item groupings were identified. Three different models were analyzed. The first (Model A) focused on EPIQ timing items (e.g., the duration of a phototoxic reaction) for participants who had a phototoxic reaction in the past 7 days. These items were completed by only participants who experienced a phototoxic reaction. The second model (Model B) included EPIQ items covering quality of life, severity, and changes since the start of the study, some of which were collected at baseline and all of which were available at follow-up assessments. The third model (Model C) assessed symptoms from the Sunlight Exposure Diary (see Additional File 1).

Eigenvalues, scree plots, and factor loadings guided the factor retention and interpretation process. Variable clustering was used to confirm the factor structure. Scales were calculated as the average of non-missing items if at least half of the items in the scale were non-missing. All scales range in value from 0 to 100, where higher scores indicate better outcomes.

#### Reliability

Internal consistency reliability is a measure of the extent to which items intended to measure the same construct produce similar results. For multi-item scales, this was assessed using Cronbach’s alpha coefficient [20], with values of ≥ 0.70 considered acceptable. Test-retest reliability, which measures the consistency of results over repeated administrations for participants whose underlying disease status does not change, was evaluated using the Shrout-Fleiss (3,1) intraclass correlation coefficient (ICC) [21], with values of ≥ 0.75 indicating good reliability. The test-retest cohort of unchanged individuals included those whose EPP/XLP status, measured by the question “Since the start of the study, how would you rate the CHANGE in your EPP?” remained unchanged between Days 43 and 71.

#### Validity

Construct validity, the extent to which the Sunlight Exposure Diary and the EPIQ correlate with other measures of similar concepts, was evaluated through convergent and divergent validity. Convergent and divergent validity was assessed by correlating items and scales from the Sunlight Exposure Diary and EPIQ with several PROMIS measures [22], including PROMIS-57 v2.1, PROMIS Short Form v2.0 - Social Isolation, and PROMIS-Neuropathic Pain Quality v2.0 scales. Additionally, a priori hypotheses were established for how the scales would correlate with each other and with the PROMIS measures. These hypothesized correlations were compared with the actual correlations from empirical calculations.

Known-groups validity was assessed by evaluating the ability to differentiate between groups known to differ based on a 2-week symptom average (dichotomized at the median of 100 due to limited variability) and baseline light tolerance (< 30 min vs. ≥ 30 min). A 2-week symptom average was calculated by first assigning numeric values to the severity responses in Question 5 of the Sunlight Exposure Diary (“Please indicate if you had any of the following symptoms on the date entered at the top of the screen. If you had the symptom, please indicate whether it was mild, moderate, or severe”). There were 12 symptoms listed; values of 1, 2, or 3 reflect severities of mild, moderate, and severe, respectively. These values were averaged over the 14-day period to compute a mean severity score, which was then reverse-scored and rescaled to a 0 to 100 scale so that higher scores indicate less severity. This analysis was cross-sectional; baseline data were used for all scales and items, except for items that ask about changes in EPP/XLP symptoms or phototoxic reactions, which were only collected at follow-up. Therefore, end-of-study (EOS) data from Day 121 were used for this scale and its items. The Student’s t-test (*p* < 0.05) was used to identify significant differences between known groups.

A refinement of the known-groups validity analysis was explored post-hoc using baseline light tolerance categories of ≤ 10 min, > 10 to < 30 min, and ≥ 30 min to assess the potential for improved differentiation. This cross-sectional analysis using data from baseline included all scales and items except EPP Overall Change and its individual items, only collected at follow-up.

#### Responsiveness

Responsiveness, the ability of a measure to detect clinically important changes, even if the changes are small [23], was evaluated using standardized effect sizes [24] (SESs) and standardized response means [25] (SRMs). Success thresholds for SES and SRM were defined as values ≥ 0.5.

#### Meaningful change

Meaningful change was assessed by computing minimal detectable change (MDC) and minimal important change (MIC), using both distribution- and anchor-based methods. For the Sunlight Exposure Diary, a 2-week average for the 2 weeks before baseline and a 2-week average leading up to the EOS visit were used as “baseline” and “end of study” in the calculations. For the EPIQ, baseline and Day 121 data were used.

The MDC, which represents the smallest change that is greater than measurement error (random fluctuation) and is considered the lower bound for defining the MIC, was calculated using distribution-based methods. The standard error of measurement [26] (SEM) was the primary approach, calculated using the baseline standard deviation and a sample reliability coefficient. A lower SEM indicates more precise scores, since any change exceeding the SEM reflects a reliable change beyond measurement error. The SES (also referred to as Cohen’s d) was also used; the SES is based upon the standard deviation at baseline. Cohen defines a value of 0.20 as a “small” effect, 0.50 as a “medium” effect, and 0.80 as a “large” effect [24].

The responsiveness statistic, another distribution-based method for estimating the MDC, was calculated as the difference in means between baseline and follow-up divided by the standard deviation of the change for the stable cohort (those with no changes on the PGI-C at the end of the study).

Anchor-based methods were used to estimate the MIC, which reflects the smallest change that is considered meaningful or important, based on clinical significance and patient perception. Questions from the PGI-C were used as anchors for the concept of interest.

Psychometric analyses were performed using SAS/STAT software, Version 15.2, in SAS Studio (SAS 9.4).

## Results

### Study population

The study included 65 participants with a baseline and at least 1 follow-up assessment on the EPIQ. Participants had a mean age of 44.8 years (SD = 13.0); the gender distribution was nearly equal, with 32 female (49.2%) and 33 male (50.8%) participants. Geographically, the majority of participants were from the US Midwest or Northeast (66.2%) (Table 1).

**Table 1:** Demographic and clinical characteristics

Characteristic	Total (*N* = 65)
Sex, n (%)	
Female	32 (49.2%)
Male	33 (50.8%)
Age in years, mean (SD)	44.8 (13.0)
Whole-blood metal-free PPIX levels, µg/L	
Mean (SD)	9335.5 (5682.1)
Median (Interquartile Range)	7790 (5250, 11,230)
Two-week interval symptom average	
Mean (SD)	93.1 (11.9)
Median	100
Missing, n (%)	4 (6.2%)
< 100, n (%)	29 (44.6%)
= 100, (%)	32 (49.2%)
Geographic region, n (%)	
US Midwest or Northeast	43 (66.2%)
US South or West Region	22 (33.8%)
Baseline Light Tolerance, n (%)	
< 30 min	22 (33.8%)
≥ 30 min	43 (66.2%)

PPIX, protoporphyrin IX

Whole-blood metal-free PPIX levels, measured in µg/L, had a mean of 9335.5 (SD = 5682.1). Twenty-two participants (33.8%) had a baseline light tolerance of less than 30 min, whereas 43 participants (66.2%) could tolerate 30 min or more.

The 2-week interval symptom average shows a mean of 93.1 (SD = 11.9) and a median of 100, with 6.2% of participants having missing data for this measure. The 2-week interval symptom average suggests that most participants had infrequent symptoms.

### Individual item review and rescaling

The individual item review revealed substantial floor effects for most items. Significant floor effects can limit the ability of single item scores to be responsive to change, suggesting that scales may provide more variability and better sensitivity.

Most participants did not experience a phototoxic reaction during the study period; therefore, response rates were low for 3 EPIQ items that inquired about characteristics and timing of phototoxic reactions.

### Exploratory factor analysis

The EFA identified 3 underlying factors in the EPIQ and 1 in the Sunlight Exposure Diary. One factor emerged from Model A (Duration of Full Reaction [DFR]), 2 distinct factors emerged from Model B (Overall Change [OC] and Overall Severity and Impact [OSI]), and 1 factor emerged from Model C (Tingling/Itching) (Table 2). Upon review, it was deemed that the Tingling/Itching factor did not represent a true latent construct and therefore its psychometric properties were not assessed.

**Table 2:** Results of exploratory factor analysis, internal consistency, and Test-Retest reliability testing

EFA Results						Reliability Results		Factor Characteristics
Factor Visits Used	Item Numbers Item Labels		Eigen- value(s)	Factor Loading Range (Variance Explained)		Cronbach’s Alpha	ICC	Floor (%); Ceiling (%)
**EPIQ**								
**Model A**,** Factor 1**: Duration of Full Reaction *Days 1*,* 43*,* 71*,* and 121 combined*	Once you started to have a full reaction, approximately HOW LONG did it take for the FULL REACTION to START TO IMPROVE? Once you started to have a full reaction, approximately HOW LONG did it take for ALL YOUR SYMPTOMS from the FULL REACTION to GO AWAY?		First = 1.34; Second < 1	0.82–0.82 (1.16)		0.86	Not reported due to data sparsity	0%:0% over all assessments
**Model B**,** Factor 1**: Overall Change *Days 43*,* 71*,* and 121 combined*	Since the start of the study, how would you rate the CHANGE in how severe your full reactions were? Since the start of the study, how would you rate the CHANGE in how severe your early warning symptoms were? Since the start of the study, how would you rate the CHANGE in your EPP? How much time are you able to now spend in sunlight (direct or indirect) without having early warning symptoms compared to the start of the study? How much time are you able to now spend in sunlight (direct or indirect) without having a full reaction compared to the start of the study?		First = 5.97	0.87–0.91 (0.77)		0.97	0.97	0%;45.3% at Day 43 (not collected at Day 1)
**Model B**,** Factor 2**: Overall Severity and Impact *Days 43*,* 71*,* and 121 combined*	In the past 7 days, how much did having EPP impact your ability to do the things you want to do? In the past 7 days, how much did having EPP impact your overall quality of life? Overall, how severe were your full reactions in the past 7 days? Overall, how severe were your early warning symptoms in the past 7 days? Overall, how severe was your EPP in the past 7 days?		Second = 1.59; Third < 1	0.59–0.81 (0.21)		0.88	0.62	0%; 4.6% at Day 1
**Sunlight Exposure Diary**								
**Model C**, Factor* 1: Tingling / Itching *Days 1*,* 43*,* 71*,* and 121 combined*	Tingling severity Itching severity		First = 0.82; Second < 1	0.64–0.64 (1.44)		Not Assessed	Not Assessed	0%;67.3% at Day 1
**Daily Daylight Tolerance Over 2-Week Intervals**								
Individual item		(Not included in EFA because it is an individual item)	NA		NA	NA	0.89	1.5%;0% at Day 1

EFA, exploratory factor analysis; EPIQ, Erythropoietic Protoporphyria Impact Questionnaire; EPP, erythropoietic protoporphyria; ICC, intraclass correlation coefficient; NA, not applicable

There was not a specified factor loading cut off in the rotated factor matrices

*Upon review, it was deemed that the Tingling/Itching factor did not represent a true latent construct and therefore its psychometric properties were not assessed

Some items did not load onto any factor, and others were treated as a standalone item (e.g., “Daily Daylight Tolerance Over Two-Week Intervals” [DDT-TWI]). Many symptom items were too sparsely populated for aggregation.

Mean scores ± SD at Days and 121, respectively, were 46.2 ± 18.3 and 49.8 ± 19.6 (DDT-TWI), 76.6 ± 14.3 and 75.0 ± 5.1 (DFR), 70.8 ± 18.3 and 93.2 ± 12.2 (OSI), and 85.8 ± 20.4 (OC; Day 121 only).

### Reliability

Most scales demonstrated strong internal consistency and test-retest reliability, indicating that the individual items and scales reliably measure the same construct and that the tool consistently produces similar results when administered multiple times to the same individuals. The Daily Daylight Tolerance Over Two-Week Intervals scale showed excellent test-retest reliability with an ICC of 0.89, and the DFR scale had solid internal consistency with a Cronbach’s alpha of 0.86 and a reasonable correlation with the total range at 0.76. The OC scale displayed outstanding reliability, with a Cronbach’s alpha of 0.97 and an ICC of 0.97. However, it was used to define the stable cohort used for ICC, so that value is artificially inflated. The OSI scale demonstrated solid reliability, with a Cronbach’s alpha of 0.88, but it had some variability in item-total correlations, and its ICC is lower (0.62) than desired (Table 2).

### Validity

Construct validity results revealed that the DDT-TWI showed poor alignment with most hypotheses, which may be a result of the use of a 2-week period (instead of a longer period), causing increased variability. The OSI hypotheses with the PROMIS held, except for 1 correlation (with the PROMIS Physical Function). The DFR and OC scales demonstrated reasonable alignment with the hypotheses, though with some variability. Detailed results can be found in Supplementary Material 3.

**Fig. 1:**
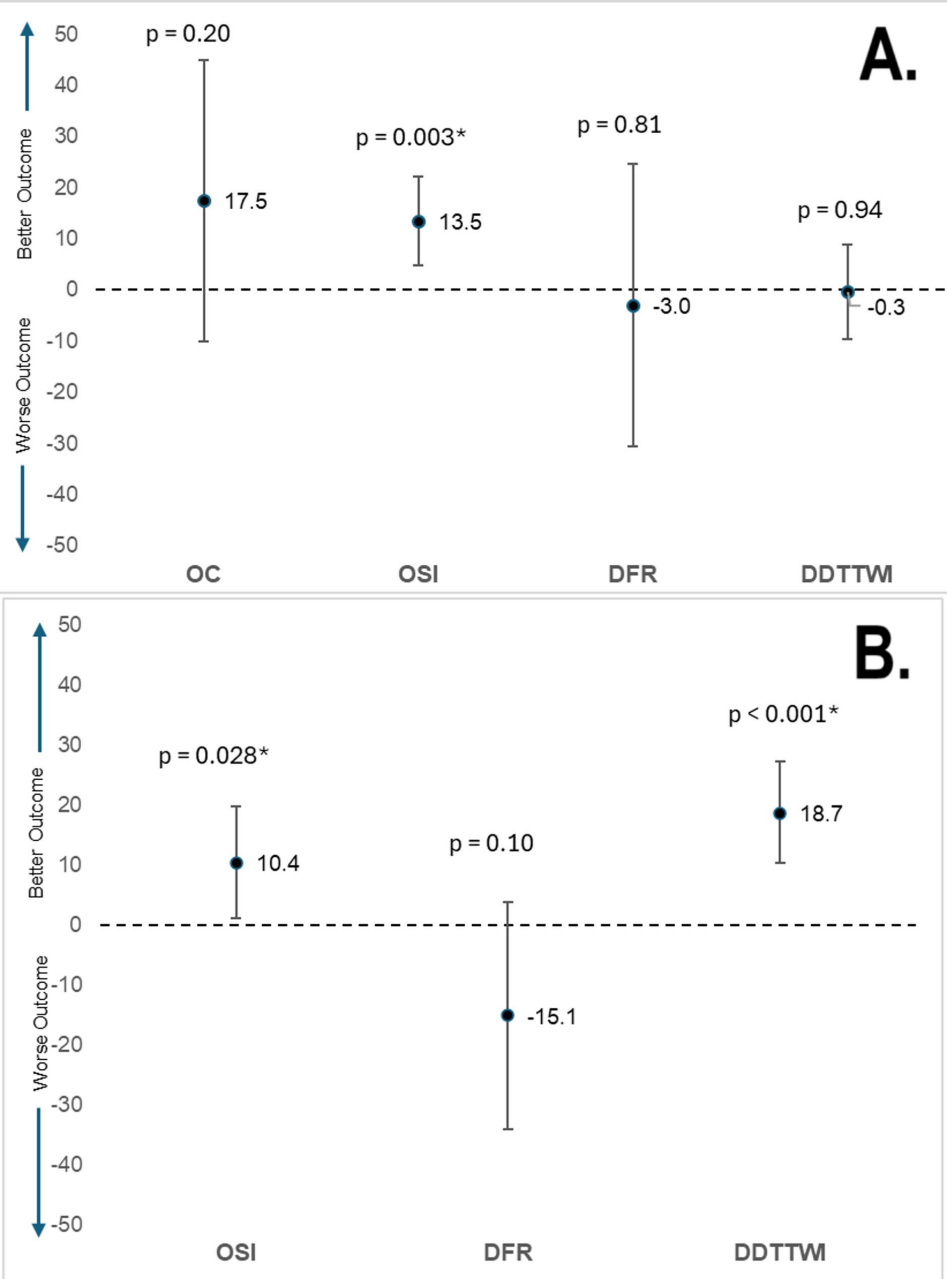
Forest Plot of Known-Groups Validity: Mean difference in (**A**) average symptom score categories and (**B**) time to prodrome categories. The symbol “*” indicates a statistically significant result using an alpha of 0.05. DDTTWI, Daily Daylight Tolerance Over Two-Week Intervals; DFR, Duration of Full Reaction; OC, Overall Change; OSI, Overall Severity and Impact

In the known-groups validity analysis, both the OSI scale and the DDT-TWI could differentiate between groups known to be different, but the OC and DFR scales could not. Figure 1 displays forest plots depicting the mean differences in scale scores between groups based on severity (average symptom score category < vs. = 100) and time to prodrome (< vs. ≥30 min) of the scales. Given that higher scores are expected for those with lower symptom severity and longer time to prodrome, a positive mean difference indicates a better outcome.

Exploratory known-groups analysis using the 3-category time-to-prodrome classification showed results similar to the 2-category analysis (not shown).

### Responsiveness and meaningful change

Meaningful change analyses were assessed using both distribution- and anchor-based results, the latter of which were challenged by low sample sizes. The meaningful detectable change for the OSI score was 6.4 points, appropriately lower than the minimal important difference of 21 to 24 points on a 0 to 100 scale. Responsiveness results suggest that the OSI scale is the most responsive, while the DDT-TWI scale is least responsive to change (Table 3).

The DDT-TWI scale showed low responsiveness, as indicated by its small SES (0.20) and SRM (0.16), alongside a negative responsiveness statistic range (-0.91 to -0.55). The DFR scale demonstrated moderate responsiveness, with an SES of -0.11 (low) and an SRM of 0.92 (high), though the responsiveness statistic is not available. The OSI scale exhibited high responsiveness, with a strong SES of 1.23 and an SRM of 0.92, supported by a positive, albeit low, responsiveness statistic range (0.06 to 0.14, Table 3).

**Table 3:** Meaningful change and responsiveness

Scale Label	MIC Range	Exploratory: Important Change Range	MDC	Responsiveness Statistic Range	SES	SRM
**Daily Daylight Tolerance Over 2-Week Intervals**	-9.2 to -0.2	6.7 to 7.2	6.1	-0.91 to -0.55	0.20	0.16
**Daily Daylight Tolerance Over 1-Month Intervals**	-0.9 to 7.1	13.3 to 13.5	Not Assessed			
**Duration of Full Reaction**	N/A	Not Assessed	5.3	NA	-0.11	0.92
**Overall Severity and Impact**	21.0 to 24.0	Not Assessed	6.4	0.06 to 0.14	1.23	0.92

ICC, intraclass correlation coefficient; MDC, minimal detectable change; MIC, minimal important change; NA, not applicable; SES, standardized effect size; SRM, standardized response mean

The standard error of measurement (SEM) was the primary approach, calculated using the baseline standard deviation and a sample reliability coefficient: Cronbach’s alpha for multi-item scales and ICC for single items

The exploratory meaningful change analyses for the DDT-TWI measures based on combined anchor categories revealed a range of 6.7 to 7.2 for the 2-week interval, whereas the 1-month interval showed a higher range of 13.3 to 13.5 (Table 3; Additional File 2). The 1-month interval provided a clearer and potentially more reliable estimate for important change compared to the 2-week interval.

### Floor and ceiling effects

High ceiling effects were noted for many items starting at Day 1, indicating that a substantial portion of participants was scoring at the upper limit of these measures.

## Discussion

The psychometric evaluation confirms the robustness of the EPIQ, particularly the OSI scale, which demonstrated strong psychometric properties in terms of reliability, validity, and responsiveness. The DFR and OC scales also exhibited acceptable psychometric properties. The DDT-TWI scale showed less consistent performance, with weaker validity and responsiveness. The 2-week interval for data collection may not have been long enough to allow for sufficient opportunities for sunlight exposure; responsiveness appeared to improve using the 1-month interval calculation. However, it should be noted that the type and intensity of symptoms can vary between individuals, and symptom severity may be affected by the amount of light exposure from a previous period (i.e., the day before) [27].

Results suggest that the PROMs are reliable and valid, supporting their use in future clinical research and relevant for assessing the experiences of individuals with EPP or XLP. These tools address the need for EPP/XLP-specific PROMs that assess the duration, severity, and impact of early warning symptoms and full phototoxic reactions, and capture impacts of EPP and XLP on well-being and HRQoL.

The study was limited by small sample sizes in some analyses, including responsiveness and meaningful change assessments, where sparse data limited the calculation of key MDC and MIC statistics for some scales, thereby weakening the conclusions regarding these measures. Small sample sizes are a common struggle in research of rare diseases [28] and pose analytic challenges. Limited variability in the known-groups analyses restricted the ability to detect meaningful differences, further affecting the robustness of the findings.

Future studies may focus on exploring a 1-month interval for daylight tolerance instead of 2 weeks, thereby increasing sample sizes to improve the robustness of responsiveness and meaningful change analyses and enhancing the variability of known groups to strengthen validity testing. Test-retest reliability should be re-evaluated using a shorter time interval between the “test” and “retest” to address observed inconsistencies, and the factor structure identified here should be confirmed in a future study with a different sample. These additional elements can further enhance the validity, reliability, and responsiveness of the measures and would provide additional support for the instruments’ continued use and utility in future research and clinical practice.

## Conclusions

The EPIQ and Sunlight Exposure Diary are reliable and valid and are relevant for assessing the experiences of individuals with EPP or XLP. This supports their use in future studies within this population.

## Supplementary Information

Below is the link to the electronic supplementary material.

Supplementary Material 1

Supplementary Material 2

Supplementary Material 3

## Data Availability

Data are not available due to the proprietary nature of the clinical trial.

## Abbreviations

DDT-TWI: Daily daylight tolerance over two week intervals
DFR: Duration of full reaction
DLQI: The dermatology life quality index
EFA: Exploratory factor analysis
EOS: End-of-study
EPIQ: The EPP impact questionnaire
EPP: Erythropoietic protoporphyria
HRQoL: Health-related quality of life
ICC: Intraclass correlation
MDC: Minimal detectable change
MIC: Minimal important change
OC: Overall change
OSI: Overall severity and impact
PGI-C: Patient global impression of change
PGI-S: Patient global impression of severity
PPIX: Protoporphyrin IX
PROM: Patient-reported outcome measure
PROMIS: Patient-reported outcomes measurement system
SD: Standard deviation
SEM: Standard error of measurement
SES: Standardized effect size
SRM: Standardized response mean
XLP: X-linked protoporphyria
