# Supplementary material for: Validation of the sunlight exposure diary and the erythropoietic protoporphyria impact questionnaire (EPIQ)
Source: Orphanet J Rare Dis. 2025 Sep 30;20:492. doi: 10.1186/s13023-025-04012-8 (PMC12486579; doi:10.1186/s13023-025-04012-8)

**Additional File 1**

Exploratory Factor Analysis Path Diagrams for Model A (a), Model B (b), and Model C (c).

(a)


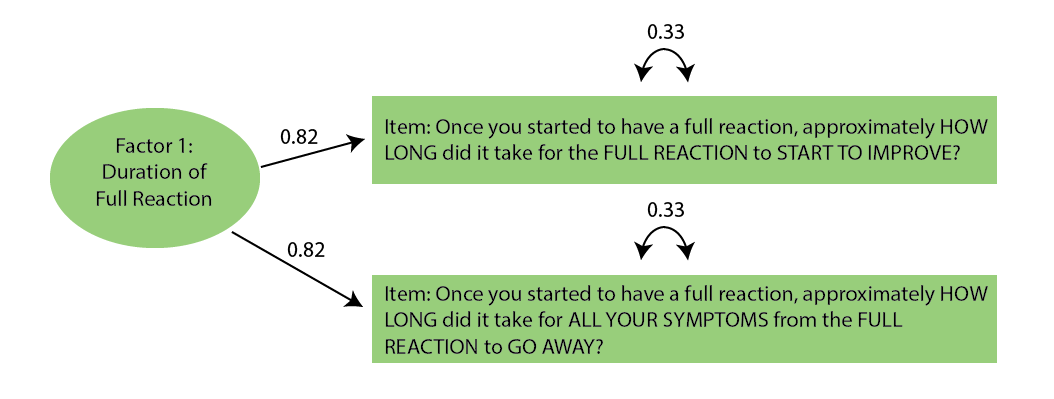


(b)


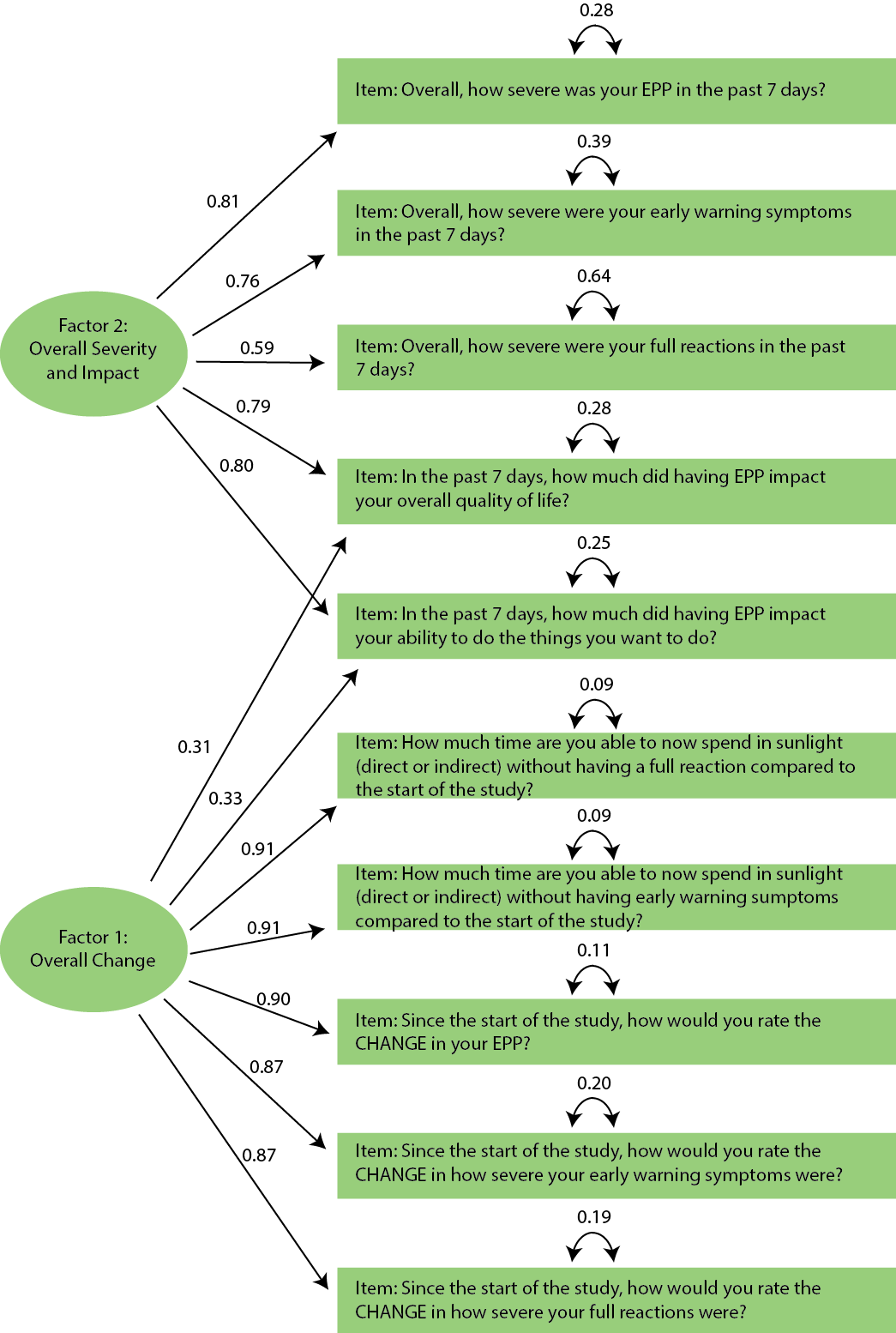


(c)


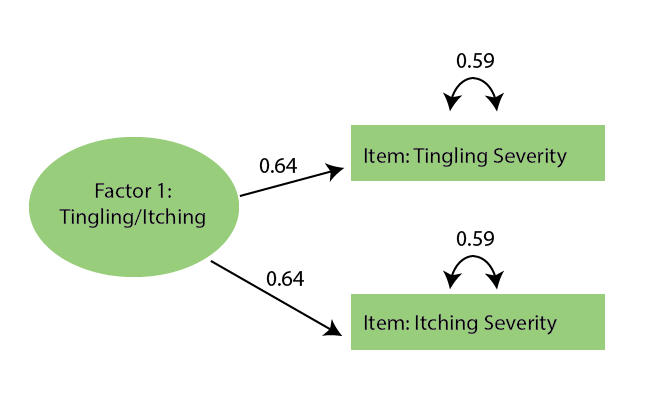

Supplement: Supplementary file 1 — Supplementary Material 1 [file 13023_2025_4012_MOESM1_ESM.docx]
