# Supplementary material for: Validation of the sunlight exposure diary and the erythropoietic protoporphyria impact questionnaire (EPIQ)
Source: Orphanet J Rare Dis. 2025 Sep 30;20:492. doi: 10.1186/s13023-025-04012-8 (PMC12486579; doi:10.1186/s13023-025-04012-8)
Supplement: Supplementary file 2 — Supplementary Material 2 [file 13023_2025_4012_MOESM2_ESM.docx]

**Additional File 2**

Daily Daylight Tolerance for (A) Two-week and (B) One-Month Intervals

A.


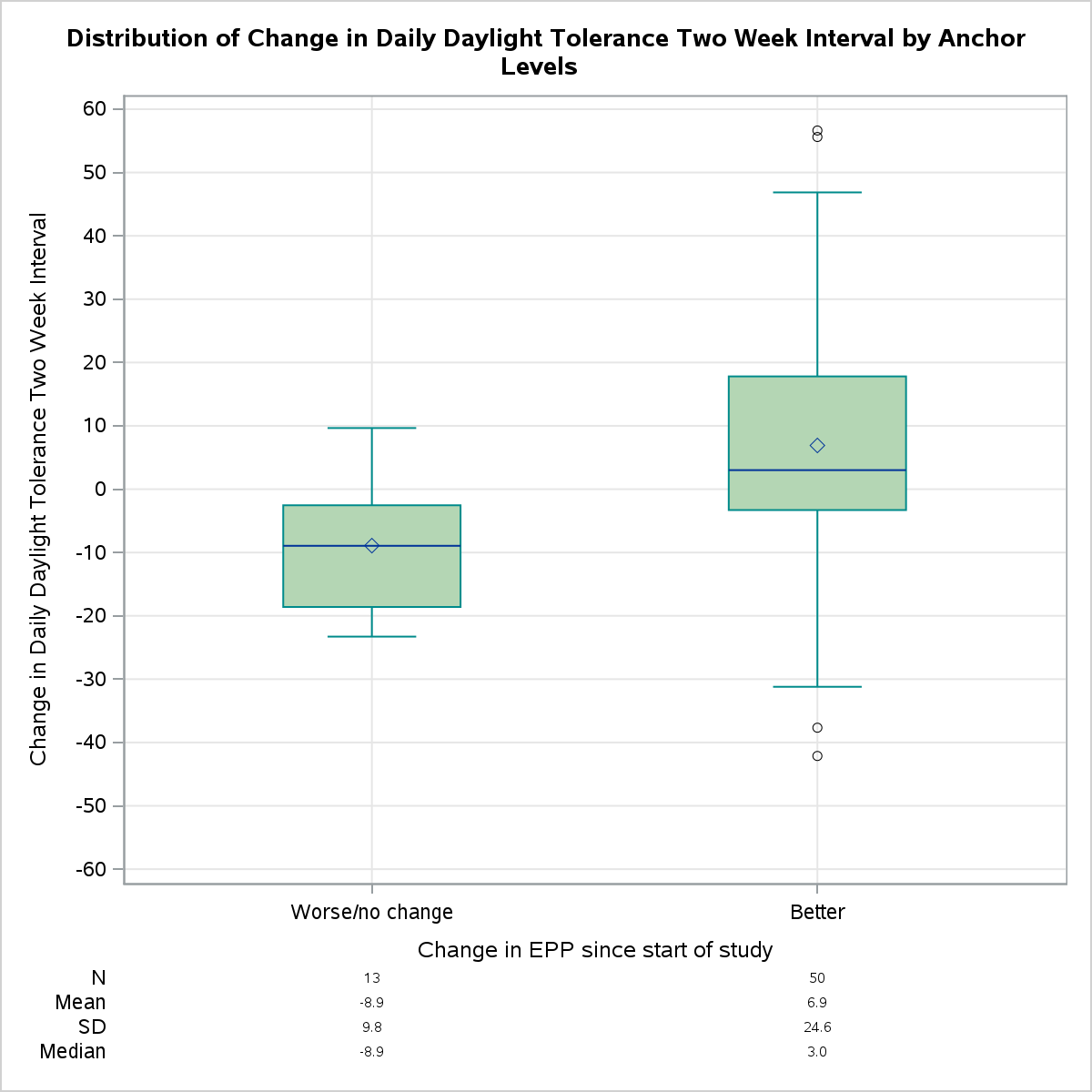


B.


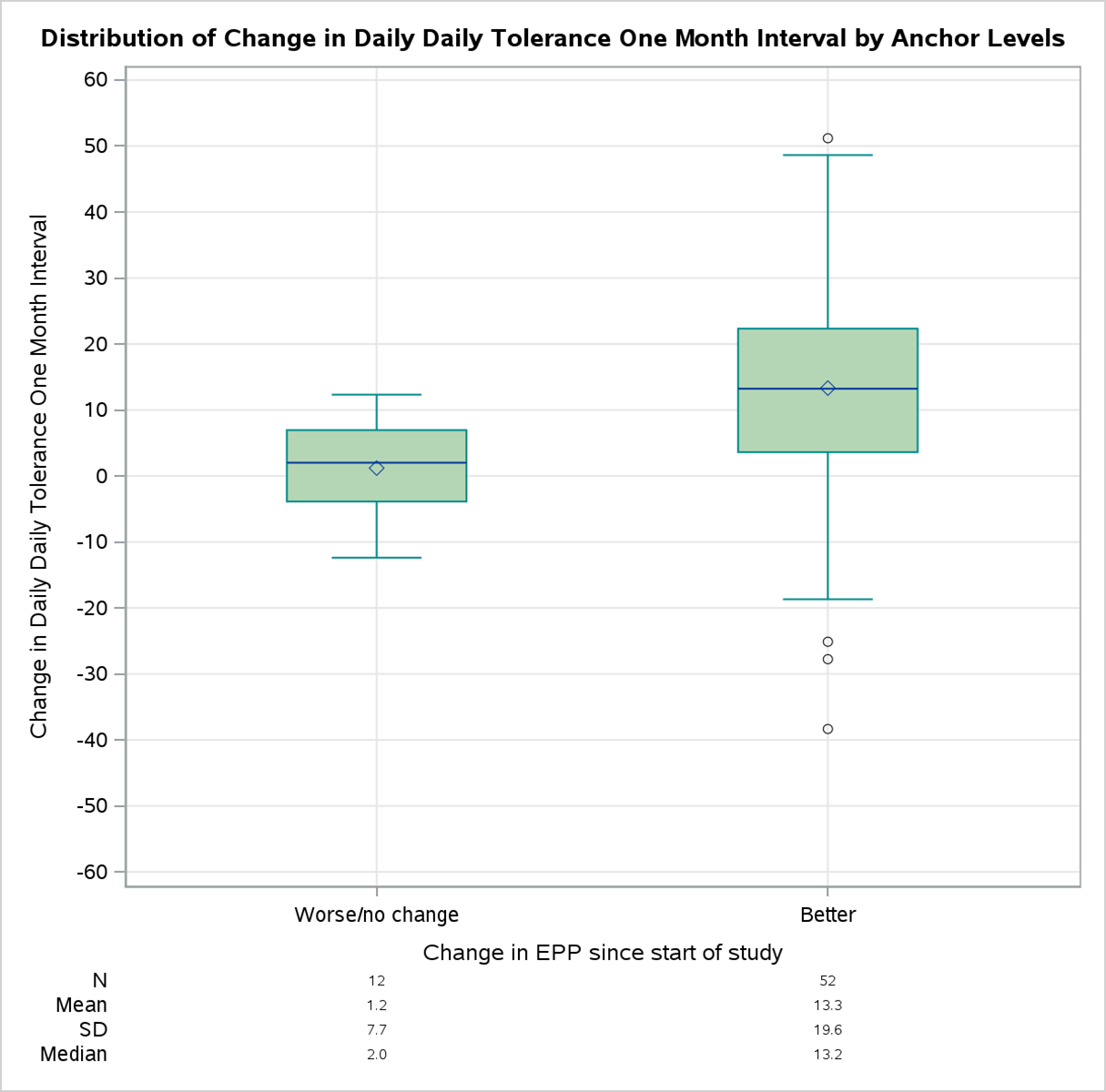


EPP, erythropoietic protoporphyria; SD, standard deviation

For each box plot, the edges of the box represent the 25^th^ and 75^th^ percentiles, with the median (bold horizontal line) and mean (diamond) inside the box. The whiskers extend to the smallest and largest data points within 1.5 times the interquartile range below/above the 25^th^/75^th^ percentiles. Circles outside of the whiskers reflect data points outside of that range.
